# Depoliticization, Colonialism, and the Imperative to Disrupt Denial: Comment on "The Rhetoric of Decolonizing Global Health Fails to Address the Reality of Settler Colonialism: Gaza as a Case in Point"

**DOI:** 10.34172/ijhpm.8761

**Published:** 2025-04-13

**Authors:** Zvika Orr, Anna C. Zielinska

**Affiliations:** ^1^Selma Jelinek School of Nursing, Jerusalem College of Technology, Jerusalem, Israel.; ^2^Department of Philosophy, Université de Lorraine, Nancy, France.; ^3^Archives Henri-Poincaré, CNRS, Nancy, France.; ^4^Sciences Po Paris, Nancy, France.

**Keywords:** Depoliticization, Colonialism, Denial, War, International Organizations, Israel/Palestine

## Abstract

This article builds on Engebretsen and Baker’s editorial to explore recent developments in medical neutrality, the depoliticization of healthcare, and political intervention in the context of the war in Gaza. We examine how international health organizations have increasingly, though insufficiently, taken a political stance, criticizing the detrimental structural forces affecting Palestinians’ life and health. Concomitantly, many Israeli healthcare professionals and organizations have shifted from a declared neutral stance to endorsing the state’s official narrative. Additionally, we analyze the connections between settler colonialism, Israeli and US policies, medicine, and international health organizations. While the discourse of decolonization provides valuable historical context for understanding the ongoing oppression of Palestinians, it often obscures critical issues, particularly the atrocity of the October 7 attack. We conclude by discussing the shift from literal denial to interpretive and implicatory denial, emphasizing the role of international health professionals and organizations in confronting these pervasive forms of denial.

## Introduction

 In their thought-provoking editorial, Eivind Engebretsen and Mona Baker use the case of the assault on health in Gaza to criticize the literature on decolonizing global health and to rethink the position of global health institutions and the systems of knowledge production that underpin the “colonial approach to the health of victims of settler colonialism” (p. 1).^1^ For instance, the authors criticize the World Health Organization (WHO) for its decades-long policy to refrain from questioning the colonial structures that perpetuate intensive assaults on the Palestinian population’s health, choosing instead to treat the aftermath of colonial violence as a humanitarian crisis.

 We propose that it would be useful to distinguish between two main aspects of this important criticism: the need to challenge depoliticized practices and address harmful structural forces, and utilizing the specific conceptual framework of settler colonialism and decolonization. After analyzing each of these aspects, we conclude by contending that international health organizations should play a decisive role in challenging the prevalent “states of denial.”^2^

## The Depoliticized Approach in Healthcare

 The first aspect of Engebretsen and Baker’s criticism is their broad criticism of the apolitical approach of global health institutions and their prevailing perspective of humanitarianism. Indeed, international health organizations often deploy depoliticized strategies for change, operating within the constraints of what Ferguson called (in a different context) “the anti-politics machine.”^3^ According to Ferguson, depoliticization does not imply the absence of political activity; rather, it is the public visibility of politics that is obscured. In this framework, the political process is effectively “whisked out of sight,” with decisions—such as the choice to refrain from intervention—still being made behind the scenes. Specifically, in organizations like WHO, political maneuvering and deliberation often take place covertly, within the realm of “soft power.”

 In many cases, the supposedly apolitical approach has had deleterious effects. For example, in the aftermath of the genocide against the Tutsi in Rwanda in 1994, international organizations provided aid to refugees in the Democratic Republic of Congo. However, due to their apolitical stance, these organizations ended up aiding the Hutu militias and genocidaires who exerted significant control in the camps and used them as bases, with long-term adverse political impacts.^4^

 Scholars have shown how in the context of Israel/Palestine, health practitioners and organizations have often disregarded state violence and other upstream political determinants of health.^5-7^ Their apolitical approach stems, in part, from the normative principle of medical neutrality. This principle seeks to establish a professional clinical space that protects both clinicians and patients from political interference and violence, ensuring impartial medical treatment.^7,8^ However, medical neutrality often unjustifiably leads clinicians to neglect political determinants of health, thereby reinforcing existing repressive systems.^7,8^ Another reason for this depoliticization is the fact that international organizations in the Occupied Palestinian Territory (oPt) are tightly controlled by Israel and are subject to Israeli-imposed restrictions.^9^ A recent example is Israel’s legal ban on the activities of United Nations Relief and Works Agency for Palestine Refugees in the Near East in Israel, which adversely affects its work in East Jerusalem.

 While we acknowledge the critique of healthcare depoliticization, we suggest that it is not universally applied. Work by WHO and critical international health organizations in the oPt has sometimes included political elements. For instance, the WHO’s reports on the right to health in the oPt reveal structural political barriers to healthcare access, as well as health-related violations such as the security interrogation and arrest of patients and their companions.^10^

 Moreover, since the writing of Engebretsen and Baker’s article, intriguing developments have occurred in the implementation and contestation of neutrality in relation to the war. Whereas Engebretsen and Baker rightly point out that WHO’s condemnations of the attacks on health in Gaza rarely name the aggressor explicitly,^1^ in recent months, international medical and humanitarian organizations have utilized increasingly political arguments to accuse Israel of its actions in Gaza explicitly. For example, in July 2024, twenty-two medical and humanitarian organizations, such as Oxfam and Médecins du Monde, published a report on the situation in Gaza, in which they cited the International Court of Justice’s decision regarding Israel’s unlawful presence in the oPt, stressing that “Israel must bring its occupation to an end, while Third States also have an obligation not to recognise as legal the unlawful presence of Israel in occupied territory, nor to render aid or assistance in maintaining the situation. Third States also have a responsibility to bring Grave Breaches of International Humanitarian Law in Gaza to an end, including, but not limited to, forcible transfer and extensive destruction of property”(p. 6).^11^

 In November 2024, the leaders of 15 United Nations and humanitarian organizations called on Israel “to cease its assault on Gaza and on the humanitarians trying to help,” using harsh terminology.^12^ In December 2024, WHO explicitly condemned Israel’s raid on a Gazan hospital, stating that “The systematic dismantling of the health system and a siege for over 80 days on North Gaza puts the lives of the 75 000 Palestinians remaining in the area at risk.”^13^

 These and many other recent documents demonstrate the growing interconnections between international legal and political endeavors, on the one hand, and healthcare and humanitarian interventions, on the other hand. This tendency of abandoning the previously dominant apolitical standpoint is a step in the right direction but insufficient given the urgent situation. It must continue and strengthen to be truly effective. It must also be translated into more effective political actions.

 A main problem is the decades-long systematic underfunding of WHO and other international organizations by rich and powerful states, which have severely weakened the organizations’ capacity and standing. The withdrawal of the United States from WHO in January 2025 is another worrisome manifestation of this structural problem. Engebretsen and Baker attributed WHO’s failing disproportionately to WHO and its policies rather than to the political-economic context in which it operates.

 Challenging the depoliticized approach alone is insufficient, as demonstrated by the discourse and praxis of most Israeli healthcare leaders and institutions in recent years. Orr and Fleming show that during the escalation of the Israeli-Palestinian conflict in May 2021, Israeli healthcare institutions and leaders called for cessation of violence between Jewish and Palestinian citizens of Israel, while largely overlooking the military campaign that was simultaneously taking place in Gaza, which was considered a controversial and political issue.^7^ Following the Hamas-led attack on October 7, 2023, many Israeli healthcare professionals and institutions adopted a pro-Israeli political stance, departing from the ethos of medical neutrality. They have defended and supported their state’s policies and official narrative concerning the war by submitting opinion articles to medical journals, reaching out to their colleagues in international associations, and responding to critical statements made by their colleagues in professional forums and on social media. At the same time, they officially silenced voices of Palestinian Israeli medical staff members who criticized Israel’s military attacks in Gaza. They also intensified efforts internationally to portray Israeli healthcare and its administration as apolitical and neutral. The narrative surrounding Israeli healthcare outside of Israel diverges from the reality within the country. Moreover, Israeli healthcare professionals who openly criticized their government usually focused solely on returning the hostages from Gaza.

 These reactions suggest that the ethos of an apolitical healthcare system is deployed unequally in Israel. Many Israeli healthcare professionals and institutions promoted a vision of the Israeli healthcare system as a neutral space, while simultaneously engaging in political affairs considered legitimate for public debate in Israel. They used concepts of neutrality to protect their authority and to silence Palestinian critical voices, while also strategically intervening in political conflict, often defending oppressive and heinous policies.

## The Conceptual Framework of Settler Colonialism

 The case of Israeli medical providers and organizations suggests that we should foster humanistic rather than nationalistic agendas in healthcare. Engebretsen and Baker promote the specific theoretical conceptualization of settler colonialism. The second aspect of their criticism of WHO and other global health organizations pertains to the fact that these organizations do not question the colonial structures underlying the assaults on Palestinians’ health. The authors call for decolonizing global health, including dismantling the current global network that supports colonialism, imperialism, and racial capitalism in varied global settings.

 Indeed, researchers have established the multiple connections between medicine and colonialism, and identified the enduring legacies of colonial medicine.^14-18^ Mukhopadhyay, for instance, meticulously shows how in Canada, the response to certain diseases was shaped by an attempt to prolong colonial control.^15^ In the context of Gaza, recent developments have made the underlying colonial logic of Israeli and international policies particularly apparent. In February 2025, US President Donald Trump unveiled a plan for the forced expulsion of approximately two million Palestinians from the Gaza Strip, proposing to “buy and own” the territory. He emphasized that the displaced Palestinians would have no right of return.^19^ Trump’s plan for ethnic cleansing was enthusiastically endorsed not only by the Israeli right-wing government but also by many Israelis who typically oppose this government. A survey revealed that 82% of Jewish Israelis support Trump’s plan.^20^ This moral distortion is alarming, even though the plan is unlikely to be realized due to Jordan and Egypt’s refusal to accept displaced Palestinians.

 This ethnic cleansing plan builds upon the ongoing destruction of the Gaza Strip, which has involved the killing of tens of thousands of Palestinians (64 260 according to a recent high estimate^21^), the flattening of entire neighborhoods and cities, the displacement of 90% of the population, and the disruption of power supplies, food delivery, and humanitarian aid. Additionally, hospitals have been attacked, Gaza’s health infrastructure has been decimated, and the water and sanitation systems have collapsed. Numerous individuals, including healthcare providers, have been detained and tortured, and at least twenty-two detainees died in Israeli custody.^22-24^

 These inhumane practices and the ethnic cleansing plan echo those of the Nakba (Arabic for catastrophe) during the 1948 war. As Palestinians have experienced throughout the current war,^24^ there is a clear connection between these two dark chapters in the history of Israeli colonialism. In 1948, more than 400 Palestinian localities were destroyed, and between 714 000 and 780 000 Palestinians^25,26^ were uprooted, denied the right to return to their homes and lands after the war.^25-28^ By November 1948, only 120 000 to 130 000 Arabs, including Palestinians and Bedouins, remained in Israel.^25^

 After the Nakba, Israel pursued its colonization project through a “strategy of domination, expansion, and control,” which Halper described as “The Matrix of Control.”^29^ This strategy encompasses “a maze of laws, military orders, planning procedures, limitations on movement, Kafkaesque bureaucracy, settlements…”(p. 94).^29^ This routine was accompanied by ongoing low-intensity warfare, as well as periodic escalations of assault on the oPt.^29,30^

 The concept of colonization is often glaringly absent in the discourse and practices of international health organizations, as well as among individual healthcare providers. As Marya and Patel argue, most physicians have unwittingly inherited a colonial worldview that detaches illness from its socio-historical contexts (p. 12).^17^ Specifically, global health scholars tend to refrain from addressing current imperial or colonial wars (p. 2).^31^ Hence, there is a need to adopt a broader historical perspective that links the colonial past and present and connects local phenomena to global processes.

 Having said that, the discourse of decolonization of Israel/Palestine tends to obscure several issues. First, scholars who employ this perspective typically overlook the atrocity of October 7. Strikingly, Engebretsen and Baker’s editorial^1^ as well as the commentaries on their editorial^9,18,31^ disregarded the Hamas-led attacks, which resulted in the killing of approximately 1200 Israelis and international civilians and the taking of 251 hostages. The narrative suggesting that this attack, while admittedly violent, was not different from other moments of violence in the recent history, frames it as a legitimate reaction to Israeli violence. Moreover, the refusal to acknowledge the trauma of October 7, genuinely lived through by Jewish and Palestinian citizens of Israel, will likely nourish the Israeli resentment and render it more difficult to reach a sustainable solution. The recognition of the suffering of Israeli and international civilians on and following October 7 by no means justifies the subsequent Israeli devastating actions. It also does not deny the asymmetry of power and the settler colonial context. The moral clarity rightly demanded by commentators^31^ must include a rejection of the immoral and unscientific disregard for Hamas’s war crimes, which have caused death and suffering to countless civilians, including peace and human rights activists like Vivian Silver and Gadi Moses.

 Additionally, the discourse of decolonization should not overlook the unique characteristics of the Israeli case. The conceptualization of Israel as a settler colonial state should engage with arguments about the Jews’ deep historical ties to Israel, as well as the lack of a mother country of which the settlers were an extension.^32^ The advocacy for decolonization should tackle, rather than ignore, these purported characteristics, which did not exist in other colonies such as Canada or Australia. Of course, these characteristics cannot justify any abuse of another people’s rights, eg, the Nakba or the contemporary killing and destruction in the oPt.

## From Denial to Action

 Claude Lanzmann’s documentary film, The Karski Report,^33^ discusses the denial and indifference of Western Allies’ prominent leaders during World War II. Lanzmann opens his film with a quotation of Raymond Aron, the French sociologist and philosopher who would later vocally oppose the French colonization in Algeria. “Someone once asked Raymond Aron—who was exiled in London at the time—if he had known about what was happening in Eastern Europe. He answered: I knew, but I didn’t believe it, and because I didn’t believe it, I didn’t know.”^33^

 Aron’s disbelieve, which led to unknowing, is a state of denial that Stanley Cohen identifies as literal (factual, blatant) denial, in which “the fact or knowledge of the fact is denied” (p. 7).^2^ In the case of Gaza, this form of denial is no longer common. Instead, there has been a shift toward two other widespread forms of denial. The first is interpretive denial, where the raw facts are not being denied but are given a different meaning from what seems apparent to others.^2^ For instance, the proposed ethnic cleansing of Palestinians was presented by Trump as “a real estate development for the future.”^19^ “By changing words, by euphemism, by technical jargon, the observer disputes the cognitive meaning given to an event and re-allocates it to another class of event” (p. 8).^2^

 The second prevalent state of denial is implicatory denial, which includes justifications, rationalizations, and evasions in which “the psychological, political or moral implications that conventionally follow” the facts are denied or minimized (p. 8).^2^ In other words, the facts are acknowledged but are not seen as psychologically disturbing or as carrying a moral imperative to act.^2,27,34^ Implicatory denial with regard to the war is evident, for example, in the mainstream discourse in Israel, which views the “flattening of Gaza” as a just, moral, and reasonable response to October 7 rather than a catastrophe. It is also evident in the scholarly critical discourse that overlooks the atrocity of October 7. A main goal of international health organizations should be disrupting the interpretive and implicatory denial.^27,28^

 To foster such transformation, international health organizations and professionals must engage in what Marya and Patel called deep medicine: “a medicine that is mindful and active in resisting colonial cosmology”(p. 22).^17^ Deep medicine in contexts of war and genocide entails being cognizant of and working to dismantle the larger political oppressive structures that have enabled and led to the current atrocities.^17^ This practice would benefit from adopting a new perspective on medicine as a labor of liberation, as Mukhopadhyay explains: “If health means a person free to walk out into the world to bring her gifts to the world, that she be a liberated being, untrammelled by illness or pain, all medicine should be liberating work, all healing a labour of liberation” (p. 5).^16^ This approach would amplify dissenting voices that have been silenced, challenge the epistemic injustice faced by Palestinians, and promote meaningful acts of solidarity with them.^6^

 In January 2025, Gadi Moses, an 80-year-old globally renowned agronomist who has trained farmers worldwide, was released from Gaza after 482 days in Hamas captivity. Israeli media reported that soon before his release, “Moses promised his captors that when peace comes, he will visit them in the Gaza Strip and teach them agriculture.”^35^ “Did he actually say that, after 482 days of agony?” we asked his niece, Efrat Machikawa, a doctoral student in medical education. A day later, Efrat called us. “Yes, Gadi confirmed it to me but corrected one mistake made by the journalists,” she said. “Gadi told me, ‘I said to the captors that I will share my knowledge with them, not teach them, for I don’t teach anyone – I only share what I’ve learned.’” If, during his time of immense suffering, Gadi not only kept striving for peace but also remained sensitive to the need for an equal relationship between Israelis and Palestinians, we cannot afford to lose hope. Healthcare providers and organizations have a moral and professional responsibility to engage in efforts to dismantle oppressive structural forces and proactively promote peace, justice, equality, and freedom for all in Israel/Palestine.

## Ethical issues

 Not applicable.

## Conflicts of interest

 Authors declare that they have no conflicts of interest.

## References

[1] Engebretsen E, Baker M (2024). The rhetoric of decolonizing global health fails to address the reality of settler colonialism: Gaza as a case in point. Int J Health Policy Manag.

[2] Cohen S. States of Denial: Knowing About Atrocities and Suffering. Polity Press; 2001.

[3] Ferguson J. The Anti-Politics Machine: Development, Depoliticization, and Bureaucratic Power in Lesotho. University of Minnesota Press; 1994.

[4] Lischer SK (2003). Collateral damage: humanitarian assistance as a cause of conflict. Int Secur.

[5] Abuelaish I, Goodstadt MS, Mouhaffel R (2020). Interdependence between health and peace: a call for a new paradigm. Health Promot Int.

[6] Lederman Z, Kayali Browne T, Kayali L, Lederman S, Orr Z (2025). Loneliness as lack of solidarity: the case of Palestinians standing alone. Bioethics.

[7] Orr Z, Fleming MD (2023). Medical neutrality and structural competency in conflict zones: Israeli healthcare professionals’ reaction to political violence. Glob Public Health.

[8] Orr Z, Jackson L, Alpert EA, Fleming MD (2022). Neutrality, conflict, and structural determinants of health in a Jerusalem emergency department. Int J Equity Health.

[9] Veronese G, Kagee A, Abu Jamei Y (2024). Confronting the colonial roots of global health inequities in Gaza: Comment on “The rhetoric of decolonizing global health fails to address the reality of settler colonialism: Gaza as a case in point”. Int J Health Policy Manag.

[10] World Health Organization (WHO). Right to Health 2017. WHO; 2018. http://www.emro.who.int/images/stories/who_right_to_health_book_for_web.pdf?ua=1&ua=1&ua=1&ua=1. Accessed August 13, 2024.

[11] Oxfam, Médecins du Monde et al. Gaza Humanitarian Snapshot #2: 13-29 July 2024. 2024. https://www.medecinsdumonde.org/app/uploads/2024/07/Copy-of-Gaza-Humanitarian-Snapshot-13.pdf. Accessed August 13, 2024.

[12] Inter-Agency Standing Committee. Statement by Principals of the Inter-Agency Standing Committee – Stop the Assault on Palestinians in Gaza and on Those Trying to Help Them. 2024. https://www.who.int/news/item/01-11-2024-statement-by-principals-of-the-inter-agency-standing-committee---stop-the-assault-on-palestinians-in-gaza-and-on-those-trying-to-help-them. Accessed February 17, 2025.

[13] World Health Organization (WHO). Kamal Adwan Hospital Out of Service Following a Raid Yesterday and Repeated Attacks Since October. WHO; 2024. https://www.who.int/news/item/28-12-2024-kamal-adwan-hospital-out-of-service-following-a-raid-today-and-repeated-attacks-since-october. Accessed February 17, 2025.

[14] Greene J, Basilico M, Kim H, Farmer P. Colonial medicine and its legacies. In: Farmer P, Kleinman A, Kim JY, Basilico M, eds. Reimagining Global Health: An Introduction. University of California Press. 2013:33-73.

[15] Mukhopadhyay B. Country of Poxes: Three Germs and the Taking of Territory. Fernwood Publishing; 2022.

[16] Mukhopadhyay B. A Labour of Liberation. Changing Suns Press; 2016.

[17] Marya R, Patel R. Inflamed: Deep Medicine and the Anatomy of Injustice. Farrar, Straus and Giroux; 2021.

[18] BlackDeer AA (2024). BlackDeer AACountering coloniality in global health: Comment on “The rhetoric of decolonizing global health fails to address the reality of settler colonialism: Gaza as a case in point”. Int J Health Policy Manag.

[19] Gritten D. Trump Says No Right of Return for Palestinians Under Gaza Plan. BBC News; 2025. https://www.bbc.com/news/articles/cn57neepx4vo. Accessed February 17, 2025.

[20] The Jewish People Policy Institute (JPPI). Majority of Israelis Support Trump’s Proposal to Relocate Gaza’s Population to Other Countries. JPPI; 2025. https://shorturl.at/zokB2. Accessed February 23, 2025.

[21] Jamaluddine Z, Abukmail H, Aly S, Campbell OM, Checchi F (2025). Traumatic injury mortality in the Gaza Strip from Oct 7, 2023, to June 30, 2024: a capture-recapture analysis. Lancet.

[22] Amnesty International. ‘You Feel Like You Are Subhuman’: Israel’s Genocide Against Palestinians in Gaza. Amnesty International; 2024. https://www.amnesty.org/en/documents/mde15/8668/2024/en/. Accessed February 17, 2025.

[23] World Health Organization (WHO). Health Conditions in the Occupied Palestinian Territory, Including East Jerusalem: Report by the Director-General. WHO; 2025. https://apps.who.int/gb/ebwha/pdf_files/EB156/B156_20-en.pdf. Accessed February 23, 2025.

[24] Human Rights Watch. “Hopeless, Starving, and Besieged”: Israel’s Forced Displacement of Palestinians in Gaza. Human Rights Watch; 2024. https://www.hrw.org/sites/default/files/media_2024/11/gaza_displacement1124web_0.pdf. Accessed February 17, 2025.

[25] Abu-Lughod JL. The demographic transformation of Palestine. In: Abu-Lughod I, ed. The Transformation of Palestine: Essays on the Origin and Development of the Arab-Israeli Conflict. Northwestern University Press. 1971:139-163.

[26] Khalidi W. All That Remains: The Palestinian Villages Occupied and Depopulated by Israel in 1948. Institute for Palestine Studies; 1992.

[27] Orr Z, Golan D (2014). Human rights NGOs in Israel: collective memory and denial. Int J Hum Rights.

[28] Golan D, Orr Z, Ershied S. Lifta and the regime of forgetting: memory work and conservation. Jerusalem Quarterly. 2013(54):69-81.

[29] Halper J. Decolonizing Israel, Liberating Palestine: Zionism, Settler Colonialism, and the Case for One Democratic State. Pluto Press; 2021.

[30] Manduca P, Chalmers I, Summerfield D, Gilbert M, Ang S (2014). An open letter for the people in Gaza. Lancet.

[31] Anonymous Anonymous (2024). Are burned babies and mass graves a global health crisis? What does decolonization got to do with it?: Comment on “The rhetoric of decolonizing global health fails to address the reality of settler colonialism: Gaza as a case in point?”. Int J Health Policy Manag.

[32] Dowty A. Is Israel a Settler Colonial State? Stroum Center for Jewish Studies, University of Washington; 2022. https://jewishstudies.washington.edu/israel-hebrew/why-israel-isnt-a-settler-colonial-state/. Accessed August 13, 2024.

[33] Lanzmann C. The Karski Report. Les Films Aleph; 2010.

[34] Orr Z, Unger S (2020). Structural competency in conflict zones: challenging depoliticization in Israel. Policy Polit Nurs Pract.

[35] Maariv Online. Unbelievable Hero: The Last Words Gadi Moses Said to the Terrorists Before He Left Captivity. Maariv; 2025. https://www.maariv.co.il/news/military/article-1168974. Accessed February 6, 2025.

